# The machine learning model based on trajectory analysis of ribonucleic acid test results predicts the necessity of quarantine in recurrently positive patients with SARS-CoV-2 infection

**DOI:** 10.3389/fpubh.2022.1011277

**Published:** 2022-11-17

**Authors:** Qi-Xiang Song, Zhichao Jin, Weilin Fang, Chenxu Zhang, Chi Peng, Min Chen, Xu Zhuang, Wei Zhai, Jun Wang, Min Cao, Shun Wei, Xia Cai, Lei Pan, Qingrong Xu, Junhua Zheng

**Affiliations:** ^1^Department of Urology, Ren Ji Hospital, Shanghai Jiao Tong University School of Medicine, Shanghai, China; ^2^Department of Health Statistics, Naval Medical University, Shanghai, China; ^3^Department of Nursing, Ren Ji Hospital, Shanghai Jiao Tong University School of Medicine, Shanghai, China; ^4^Department of Obstetrics and Gynecology, Ren Ji Hospital, Shanghai Jiao Tong University School of Medicine, Shanghai, China; ^5^Department of Interventional Oncology, Ren Ji Hospital, Shanghai Jiao Tong University School of Medicine, Shanghai, China; ^6^Department of Emergency, Longhua Hospital Affiliated to Shanghai University of Traditional Chinese Medicine, Shanghai, China; ^7^Department of Information Center, Ren Ji Hospital, Shanghai Jiao Tong University School of Medicine, Shanghai, China; ^8^BSL-3 Laboratory of Fudan University, Shanghai, China; ^9^Department of Rheumatology, Ren Ji Hospital, Shanghai Jiao Tong University School of Medicine, Shanghai, China; ^10^Department of Orthopedics, Ren Ji Hospital, Shanghai Jiao Tong University School of Medicine, Shanghai, China

**Keywords:** SARS-CoV-2, recurrently positive, nucleic acid test, viral load, virus isolation, infectivity

## Abstract

**Background:**

SARS-CoV-2 patients re-experiencing positive nucleic acid test results after recovery is a concerning phenomenon. Current pandemic prevention strategy demands the quarantine of all recurrently positive patients. This study provided evidence on whether quarantine is required in those patients, and predictive algorithms to detect subjects with infectious possibility.

**Methods:**

This observational study recruited recurrently positive patients who were admitted to our shelter hospital between May 12 and June 10, 2022. The demographic and epidemiologic data was collected, and nucleic acid tests were performed daily. virus isolation was done in randomly selected cases. The group-based trajectory model was developed based on the cycle threshold (Ct) value variations. Machine learning models were validated for prediction accuracy.

**Results:**

Among the 494 subjects, 72.04% were asymptomatic, and 23.08% had a Ct value under 30 at recurrence. Two trajectories were identified with either rapid (92.24%) or delayed (7.76%) recovery of Ct values. The latter had significantly higher incidence of comorbidities; lower Ct value at recurrence; more persistent cough; and more frequently reported close contacts infection compared with those recovered rapidly. However, negative virus isolation was reported in all selected samples. Our predictive model can efficiently discriminate those with delayed Ct value recovery and infectious potentials.

**Conclusion:**

Quarantine seems to be unnecessary for the majority of re-positive patients who may have low transmission risks. Our predictive algorithm can screen out the suspiciously infectious individuals for quarantine. These findings may assist the enaction of SARS-CoV-2 pandemic prevention strategies regarding recurrently positive patients in the future.

## Introduction

Since the late February 2022, an outbreak of severe acute respiratory syndrome coronavirus 2 (SARS-CoV-2) infection have swept Shanghai. It is estimated that from March 1 to June 10, 626,948 cases were identified, consisting 58,052 (9.26%) symptomatic cases and 588 (0.09%) death due to SARS-CoV-2 infection, according to the report from Shanghai Municipal Health Commission.

The government has initiated a series of strict and comprehensive pandemic control strategies, including the lockdown of the whole city; home-based surveillance for viral nucleic acid and antigen; and the establishment of temporary shelter hospitals for the quarantine of infected individuals, just to name a few (1). Undoubtedly, these drastic actions effectively cut off the transmission route and reduced the emerging infected cases. However, starting from May 2022, the phenomenon of an increasing number of people with recurrently positive cycle threshold (Ct) values on real-time reverse transcriptase-polymerase chain reaction (RT-PCR) assays have brought to our attention.

According to a systematic review, the incidence of recurrent SARS-CoV-2 positivity was 14.8% (2). Note that, this number is highly variable across studies, due to the different sampling approaches, hospital discharge criteria, definitions of positive nucleic acid tests and durations between hospital discharge to recurrence (2–11). Several studies have investigated the incidence rate, clinical characteristics, potential reasons and risk factors of detecting recurrently positive nucleic acid test (4–10, 12). Although most of these studies indicated that re-positive individuals are usually mildly symptomatic, with a low viral load and little risk of transmission, contradictory findings on a few recurrently cases with culturable virus have been reported (13–16). To this end, a series of concerning public health issues remain, i.e., do those patients really should be quarantined; who may pose a threat to infect the others; and who should be safe on self-monitoring?

Due to the lack of authenticated response to the above-mentioned concerns, the current pandemic prevention policy requires that anyone with recurrently positive nucleic acid test (Ct value<35) should all be readmitted to the shelter hospital for quarantine, regardless of their clinical symptoms, chest imaging manifestations and infectious potentials. While this action may indeed prevent the virus from further transmission, it considerably changes the living environment and lifestyle of both patients and their families, raising potentially psychological conditions, including sleep disorders, anxiety and depression etc. (17, 18).

We hypothesize that a predominant number of patients with recurrently positive findings on nucleic acid tests are likely to be noninfectious, and therefore, may not need mandatory quarantine. To substantiate our theory, we conducted comprehensive investigations of recurrently positive patients, including the demographic and epidemiologic features, clinical presentations, laboratory test parameters, dynamic viral RNA level variations and virus isolation. We also provided machine learning models to discern individuals who would be safe on self-monitoring, in order to avoid unnecessary quarantine.

## Participants and methods

### Study design and participants

We conducted a prospective observational study in a shelter hospital temporarily built at the Shanghai New International Expo Center, investigating patients with recurrently positive RT-PCR results after recuperated from the initial SARS-CoV-2 infection. With a capacity of over 14 thousand beds, our shelter hospital is designated by the government to admit recurrently positive patients across all areas of Shanghai, as well as those came from the neighboring cities. Free food, daily necessities, medical supplies and disease consultant were available to all of our patients.

On each day between May 12 through June 10, 2022, we carefully screened for patients, aged between 16 and 80, who have recovered (defines as at least two consecutive negative RT-PCR results with a 24-hour interval; no fever for 3 continuous days; and with no or mild respiratory symptoms) from the previous SARS-CoV-2 infection, but with recurrently positive RT-PCR findings several days after discharge. For each subject, nasopharyngeal swab sample was achieved with standard technique, and a positive reading was defined as the Ct value less than 35 during RT-PCR assay. Upon the detection of re-positivity, the patients were sent to our shelter hospital on the next day for quarantine. Instead of taking any antiviral agents or steroids, all patients were routinely prescribed Chinese medicine (Lianhuaqingwen granules, 6 g, thrice daily), with the purpose of regulating immune function. We excluded patients with severe symptoms and critical conditions, including dyspnea, hypoxemia, septic shock, acute respiratory distress syndrome, multiple organ dysfunction syndrome, cardiovascular and cerebrovascular accident etc. Those who refused to sign the informed consent, or failed to provide reliable epidemiologic and demographic information was deemed ineligible to participate.

This study has been approved by the ethics committee boards of Ren Ji Hospital (approval number: KY2022-114-B). The principles of the Declaration of Helsinki and Good Clinical Practice were complied with. This study was subjected to the STROBE reporting guidelines. All patients were required to sign the informed consent prior to recruitment.

### Data collection

At the time of admission, the demographic and epidemiologic data was collected by well-trained healthcare professionals using a unified registration form and consolidated standard. We also elaborately investigated the possible risk of viral transmission due to recurrence and the status of patients' close contacts, by asking: “where did you live after the previous hospital discharge?”, “was there anyone close to you just turned positive during the period staying together?”. It should be mentioned that the whole city was under lockdown and all citizens were restricted in their accommodations during the studying period. Therefore, if a close contact newly turned positive, it could be probable that the infection was attributed to the recurrent subjects.

### Nucleic acid tests

Nasopharyngeal swab specimens were acquired from all participants by trained nurses, staring from day 1 after admission and then each following day until criteria of recovery was reached. To detect the amount of SARS-CoV-2 RNA, RT-PCR analysis was performed by a sole clinical laboratory (Shanghai ZJ Bio-Tech Co., Ltd.) using a commercially available kit (Zhi Jiang, Shanghai, China) which is approved by the China Food and Drug Administration. The determination of positive is the Ct value less than 35 on either open reading frame (ORF) and/or nucleocapsid protein (N) genes.

### Laboratory tests

Upon approval from the patients, blood tests were done on the first day morning after admission, and hematological parameters were analyzed to reflect the features of blood cells (whole blood count); liver and kidney functions; infection and immune status (lymphocyte subpopulation and procalcitonin); and metabolism and nutrition condition (albumin, glucose, 25-hydroxyvitamin D and parathyroid hormone) based on several previous publications (19–25). All serum samples were analyzed by the clinical laboratory of Ren Ji Hospital.

### Virus isolation

Twenty-two subjects were randomly selected using computer generated randomization list, and a separate nasopharyngeal swab specimen was taken from each of them on day 1 after admission for the purpose of virus isolation. All samples were transferred to the BSL-3 laboratory of Fudan University (Shanghai, China) at 4°C within 6 h. The culture medium for vero-E6 cells contains 500 ml of Dulbecco's modified eagle medium, 100 U/ml of penicillin, 100 ug/ml of streptomycin and 10% fetal bovine serum (the concentration was 2% for maintenance medium for cell culture). All cells were tested to exclude contamination and were confirmed by morphological evaluation under microscopy. Similar to the previously described protocol for virus isolation, vero-E6 cells were plated to 80% confluency in 96-well plates (26). The specimens were seeded with cells and cultured at 37°C for 1 h. After washed with Hank's solution for 1–2 times, 3 ml of maintenance medium was added to each well. The cells were incubated at 37°C for 24h, followed by the observation of cytopathogenic effects each day. After 6 consecutive days, the cell suspension was harvested for quantitative RT-PCR to detect the RNA level of SARS-CoV-2.

### Statistical analysis

The group-based trajectory model (GBTM) was used to identify Ct value trajectories of the included patients (27). The Ct value at each timepoint was defined as the lower one between ORF and N gene during nucleic acid test. The longitudinal Ct values were fitted by a censored normal model with polynomial function of time. To identify the optimal model, 2–6 number of groups with up to three polynomial order were considered as the alternative models. All possible combinations of the alternative models were checked. The Bayesian information criterion and Akaike information criterion were used to judge the optimal model. Some other indicators were used to determine the groups of GBTM during statistical process, including the average posterior probability (>0.7), odds of correct classification (>5), the consistency between proportion assigned to group and probability of group membership, the minimum group size (>5%) and *P*-value of the highest polynomial coefficient within each group (<0.05) (27).

For the prediction models, records of included patients were divided at random, with 75% for training and 25% for testing. Recursive feature elimination (RFE) was used to select the most relevant features. We employed three machine learning algorithms to develop models, including logistic regression (LR), naive Bayes (NB) and neural network (NNET). Initially, we conducted internal validation on the development sets to quantify optimism in the predictive performance and evaluate stability of the prediction model. Cross-validation resampling technique with 100 iterations was used to evaluate the internal validity for each model. All the models were assessed in multiple dimensions regarding their model performance. The median and 95% confidence intervals of area under the curve (AUC) were calculated, where an AUC value of 1.0 means perfect discrimination and 0.5 represents no discrimination. The comparisons of epidemiologic, demographic and laboratory data were made using Chi-square test for categorical variables, and *t-*tests or Wilcoxon rank sum tests for continuous variables. The GBTM was performed by SAS 9.4 and Stata/SE 15.1 (28, 29). The prediction models were implemented by the R *caret* package.

## Results

**Figure 1:**
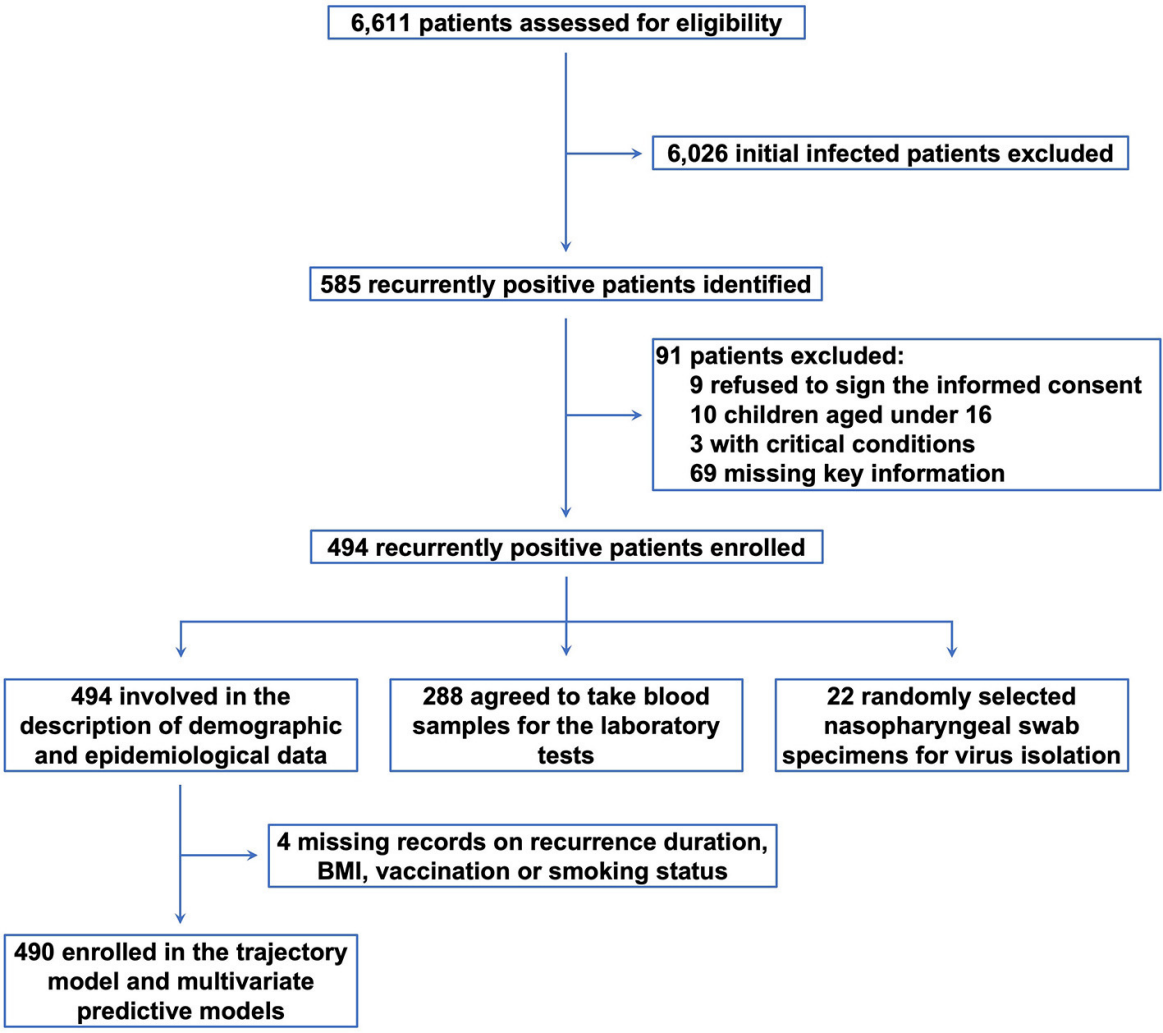
The study diagram. From May 12 through June 10, 2022, we identified 6,611 patients with SARS-CoV-2. After excluding 6,026 patients with initial infection and 91 ineligible participants, 494 subjects were enrolled for the analysis.

From May 12 through June 10, 2022, we admitted a total of 6,611 patients to our shelter hospital (Figure 1). During the epidemiological surveys, 585 patients with recurrently positive nucleic acid test results were identified. Among them, we excluded 91 subjects, including 9 refused to participate; 10 children under 16; 3 with severe symptoms and critical systematic conditions; and 69 with incomplete documentation. Subsequently, 494 subjects were enrolled for the analysis.

### The demographic and epidemiologic characteristics

We suggested that there were 62.75% of patients aged below 50, with male took up 64.37% of the total recruitment, 46.56% reported overweight or obese, and 32.99% current smokers (Table 1). The majority were vaccinated and 83.91% had taken at least 2 injections of vaccines. About 24.49% reported systematic comorbidities, there into, hypertension was the most prevalent, followed by diabetes and basic respiratory diseases.

**Table 1:** Demographic and epidemiologic characteristics.

**Variables**	**Total (*n =* 494)**	**Rapid recovery** **group (*n =* 415)**	**Delayed recovery** **group (*n =* 79)**	***P-*** **value^#^**
Age, *n* (%)				0.793
≤35	169 (34.21)	144 (34.70)	25 (31.65)	
>35 and ≤50	141 (28.54)	119 (28.67)	22 (27.85)	
>50	184 (37.25)	152 (36.63)	32 (40.51)	
Male, *n* (%)	318 (64.37)	268 (64.58)	50 (63.29)	0.928
BMI, *n* (%)				0.841
<18.5	26 (5.26)	22 (5.30)	4 (5.06)	
≥18.5 and <24	235 (47.57)	199 (47.95)	36 (45.57)	
≥24 and <28	174 (35.22)	145 (34.94)	29 (36.71)	
≥28	56 (11.34)	47 (11.33)	9 (11.39)	
Smoking habit, *n* (%)	162 (32.99)	136 (32.93)	26 (33.33)	1.000
Vaccination, *n* (%)				0.540
None	62 (12.63)	51 (12.35)	11 (14.10)	
1	17 (3.46)	15 (3.63)	2 (2.56)	
2	158 (32.18)	138 (33.41)	20 (25.64)	
3	254 (51.73)	209 (50.61)	45 (57.69)	
Comorbid conditions				
None	373 (75.51)	324 (78.07)	49 (62.03)	0.004^*^
Hypertension, *n* (%)	65 (13.16)	46 (11.08)	19 (24.05)	0.003^*^
Diabetes mellitus, *n* (%)	21 (4.25)	15 (3.61)	6 (7.59)	0.125
Respiratory diseases, *n* (%)	11 (2.23)	8 (1.93)	3 (3.80)	0.394
Others, *n* (%)	21 (4.25)	17 (4.10)	4 (5.06)	0.759
Ct value at recurrence	32.00 (30.00,33.00)	32.00 (30.00,33.00)	27.00 (24.00,30.00)	<0.001^*^
<30, *n* (%)	114 (23.08)	60 (14.46)	54 (68.35)	<0.001^*^
Recurrence duration (days)^†^	11.00 (7.00,20.00)	11.00 (7.00,20.00)	10.00 (7.00,16.75)	0.243
<7, *n* (%)	15 (3.04)	11 (2.65)	4 (5.06)	0.238
≥7, <14, *n* (%)	269 (54.45)	223 (53.73)	46 (58.23)	
≥14, *n* (%)	207 (41.90)	179 (43.13)	28 (35.44)	
Symptom rate (initial infection), *n* (%)	378 (76.52)	313 (75.42)	65 (82.28)	0.241
Symptom rate (recurrence), *n* (%)	137 (27.96)	108 (26.21)	29 (37.18)	0.066
Cough persistent over 2 weeks, *n* (%)	138 (28.11)	107 (25.91)	31 (39.74)	0.018^*^
Hospital stay (initial infection) (days)	8.00 (6.00,12.00)	8.00 (6.00,13.00)	7.00 (5.25,10.00)	0.076
Hospital stay (recurrence) (days)	4.00 (4.00,4.00)	4.00 (4.00,4.00)	5.00 (5.00,6.00)	<0.001^*^
Living status after previous discharge				0.016^*^
Solitary living, *n* (%)	43 (8.76)	39 (9.44)	4 (5.13)	
Home-based living, *n* (%)	382 (77.80)	326 (78.93)	56 (71.79)	
Grouped living, *n* (%)	66 (13.44)	48 (11.62)	18 (23.08)	
Close contacts infection during recurrence, *n* (%)^∧^	47 (9.57)	2 (0.48)	45 (57.69)	<0.001^*^

† Defines as the duration between the previous hospital discharge and recurrent nucleic acid test reports.

∧ Patients reported solitary living were excluded during the calculation.

# The comparisons between patients in rapid recovery group and delayed recovery group.

* Indicates statistically significant difference.

The median duration between the previous hospital discharge and recurrent nucleic acid test reports was 11 days (96.35% had an at least 7-day interval), and the median Ct value at recurrence was 32 (with 23.08% below 30). Note that, the hospitalization period was dramatically reduced during the second admission due to recurrence. Besides, comparing with the clinical presentations during initial infection, the proportions of overall symptomatic individuals and each detailed symptom category were remarkably lower at recurrence (Supplementary Figure 1). The investigations on subjects living with their families at home (77.80%) or in group dormitories (13.44%), uncovered a possible infection rate of (9.57%) in their close contacts during the period living together.

### The comparisons between the two groups with distinct Ct value recovery pattern

Noteworthily, as high as 84.01% of the recurrently positive subjects achieved two consecutive negative results on the first and second nucleic acid tests after admission (rapid recovery group). In contrast, 15.99% presented sluggish or fluctuated Ct value with at least one positive result during the first two tests in the hospital (delayed recovery group). The comparisons between these two groups demonstrated that the subjects in delayed recovery group had significantly higher incidence of comorbidities, particularly hypertension (*P* = 0.004 and 0.003, respectively); lower Ct value at recurrence (*P* < 0.001); more likely to have persistent cough symptom (*P* < 0.018); and more frequently reported close contacts infection (*P* < 0.001) compared with that in rapid recovery group.

### The group-based trajectory model analysis

**Figure 2:**
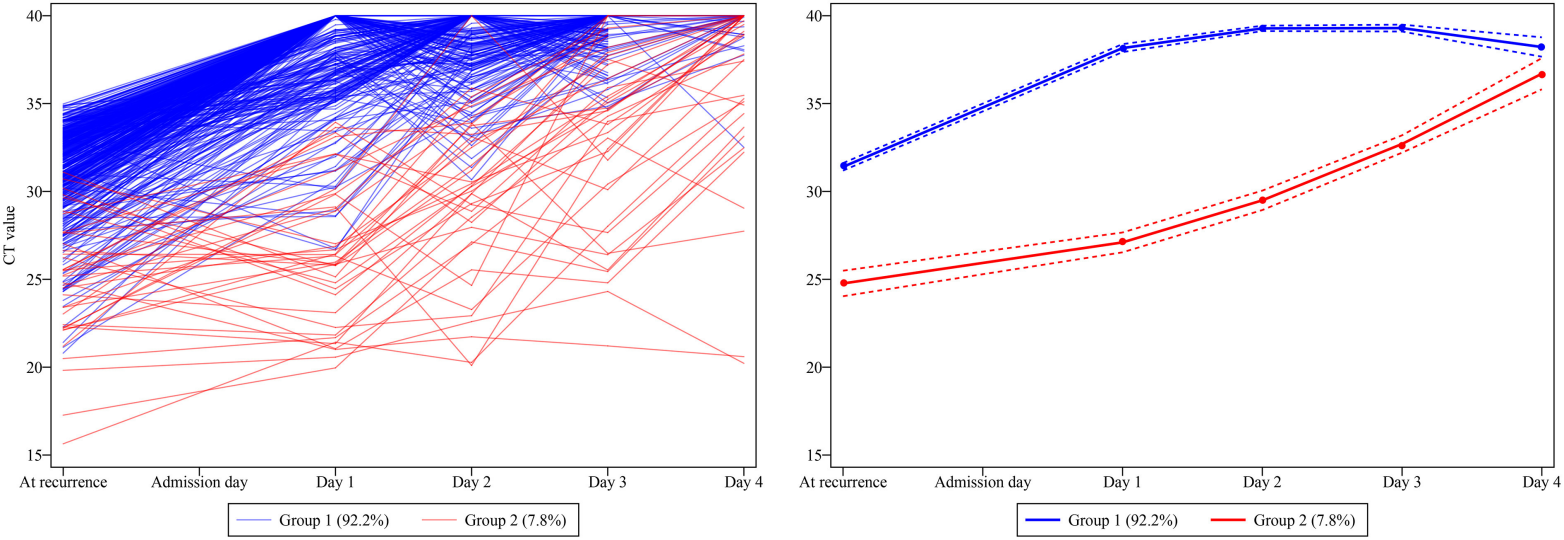
The development of trajectory groups based on the dynamic Ct value traces. Two Ct value trajectories were distinguished, namely group 1 (92.2% [452 of 490]), demonstrates a higher baseline Ct value which promptly and persistently turns to negative from day 1 after admission; and group 2 (7.8% [38 of 490]), demonstrates a lower baseline Ct value which steadily and wavily climbs back to normal after day 3.

Two trajectories were distinguished by the GBTM analysis (Figure 2), i.e. group 1 (92.2% [452 of 490]) demonstrates a higher baseline Ct value (31.93[30.28, 33.08]) which promptly and persistently returns to negative at day 1 after admission (resembles the rapid recovery group); and group 2 (7.8% [38 of 490]) demonstrates a lower baseline Ct value (25.21[22.30, 27.63]) which steadily and wavily climbs back to normal after day 3 (resembles the delayed recovery group). Echoing the comparisons between the rapid and delayed recovery group, patients in group 2 demonstrated significantly higher incidence of overall comorbidities (*P* = 0.007), hypertension (*P* < 0.001) and diabetes mellitus (*P* = 0.017); more symptoms, especially persistent cough (*P* = 0.043 and 0.047, respectively); longer hospitalization (*P* < 0.001); and more frequently reported close contacts infection (*P* < 0.001) in contrast with that in group 1 (Table 2). In addition, the abnormal rate of each laboratory test parameter between the two groups was compared (Supplementary Table 1), suggesting little notably difference, except for more frequent abnormal outcomes on percentage of monocyte (*P* < 0.001), C-reaction protein (*P* = 0.036) and serum amyloid A (*P* = 0.011) in group 2, possibly reflecting a more pronounced immune response in patients with delayed recovery of Ct value.

**Table 2:** The comparisons between the two trajectory groups.

**Variables**	**Total (*n =* 490)**	**Group 1 (*n =* 452)**	**Group 2 (*n =* 38)**	***P*** **-value^#^**
Age, *n* (%)				0.5555
≤35	168(34.29)	152(33.63)	16(42.11)	
>35 and ≤50	138(28.16)	128(28.32)	10(26.32)	
>50	184(37.55)	172(38.05)	12(31.58)	
Gender, *n* (%)				0.5757
Male	317(64.69)	294(65.04)	23(60.53)	
Female	173(35.31)	158(34.96)	15(39.47)	
BMI, *n* (%)				0.2228
<18.5	13(4.10)	11(3.81)	2(7.14)	
≥18.5 and <24	148(46.69)	138(47.75)	10(35.71)	
≥24 and <28	117(36.91)	107(37.02)	10(35.71)	
≥28	39(12.30)	33(11.42)	6(21.43)	
Smoking habit, *n* (%)	162(33.06)	148(32.74)	14(36.84)	0.6060
Vaccination, *n* (%)				0.9296
None	62(12.65)	56(12.39)	6(15.79)	
1	17(3.47)	16(3.54)	1(2.63)	
2	157(32.04)	145(32.08)	12(31.58)	
3	254(51.84)	235(51.99)	19(50.00)	
Comorbid conditions, *n* (%)				
None	372(75.92)	350(77.43)	22(57.89)	0.0068^*^
Hypertension	65(13.27)	53(11.73)	12(31.58)	0.0005^*^
Diabetes mellitus	21(4.29)	16(3.54)	5(13.16)	0.0174^*^
Respiratory diseases	11(2.24)	10(2.21)	1(2.63)	0.5924
Others	21(4.29)	19(4.20)	2(5.26)	0.6733
Symptom rate (initial infection), *n* (%)	374(76.33)	344(76.11)	30(78.95)	0.6923
Symptom rate (recurrence), *n* (%)	137(27.96)	121(26.77)	16(42.11)	0.0431^*^
Cough persistent over 2 weeks, *n* (%)	138(28.16)	122(26.99)	16(42.11)	0.0467^*^
Recurrence duration	11.00(7.00,20.00)	11.00(7.00,20.00)	11.00(7.00,17.00)	0.7362
Hospital stay (initial infection)	8.00(6.00,12.00)	8.00(6.00,12.00)	7.00(5.00,10.00)	0.1948
Hospital stay (recurrence)	4.00(4.00,4.00)	4.00(4.00,4.00)	6.00(5.00,6.00)	<0.0001^*^
Ct value at recurrence	31.64(29.70,33.00)	31.93(30.28,33.08)	25.21(22.30,27.63)	<0.0001^*^
<30, *n* (%)	136(27.76)	101(22.35)	35(92.11)	<0.0001^*^
≥30, *n* (%)	354(72.24)	351(77.65)	3(7.89)	
Close contacts infection during recurrence, *n* (%)	47(9.59)	12(2.65)	35(92.11)	<0.0001^*^

# The comparisons between patients in group 1 and group 2.

* Indicates statistically significant difference.

### The development and validation of machine learning model

**Figure 3:**
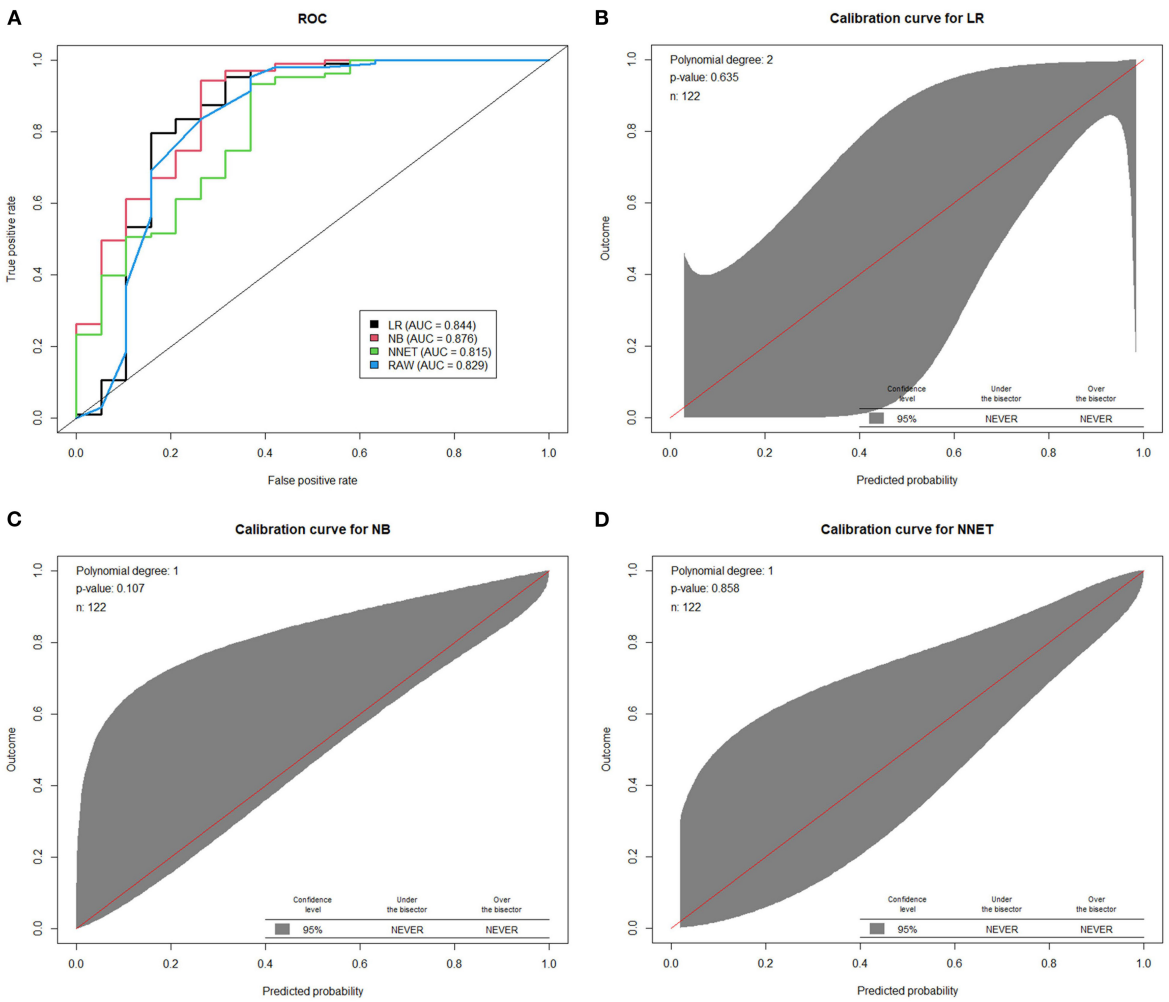
The performance of machine learning models in the validation cohort. **(A)** Area under the curve of receiver operating characteristic curve by prediction models in the validation cohort. The calibration curves of logistic regression model **(B)** random forest model **(C)** and artificial neural network model **(D)** were presented. LR, logistic regression; BN, naive Bayes; NNET, artificial neural network; RAW, only Ct value at recurrence was included in the model.

Machine learning models were developed and validated to predict the presentation of two consecutive negative nucleic acid test results immediately after admission (represents the rapid recovery feature). Fifteen predictors were extracted from the database, and 5 most important predictors (Ct value at recurrence, recurrence duration, hypertension, vaccination status and persistent cough over 2 weeks) were eventually selected using the RFE algorithm. Within the training set, the LR, NB, NNET and RAW (consisting Ct values at recurrence only) models were trained. The testing set obtained AUCs of 0.844, 0.876, 0.815, and 0.829, respectively (Figure 3A and Supplementary Table 2). Comparatively, the NB model shows the highest predictive performance among these models (AUC 0.876, 95% CI: 0.805–0.929). The calibration curves (Figures 3B–D) showed that all models performed quite well (*p* > 0.05). Additionally, a visualized and publicly accessible online calculator based on the NNET model was built (https://pengchi2009.shinyapps.io/Predict_negative/). The web server can generate an estimated negative probability by entering the covariates of the prediction model. Patients with a probability over 0.5 may demonstrate rapid Ct value recovery feature and little transmission risk, so that quarantine might be avoided.

### The virus isolation

To further evaluate the infectivity of the recurrently positive patients, virus isolation was performed on specimens from 22 randomly selected subjects, whose Ct value was ranged from 26 to 34 (Table 3). No cytopathogenic effect was observed during cell culture. Negative virus isolation results were f ound in all samples, as corroborated by testing SARS-CoV-2 RNA in the culture supernatants.

**Table 3:** Characteristics of randomly selected patients for virus isolation.

**Patients**	**Gender**	**Age (years)**	**BMI** **(kg/m^2^)**	**Vaccination** **(shots)**	**Comorbid** **conditions**	**Respiratory** **symptoms**	**Fever**	**Close contacts** **infection**	**Recurrence** **duration (days)**	**Ct value at recurrence**	**Virus isolation**
P1	M	33	24.91	3	/	Yes	/	/	11	28.51	Negative
P2	F	22	18.82	3	/	/	/	/	7	30.12	Negative
P3	M	49	23.03	3	/	Yes	/	/	7	31.11	Negative
P4	M	22	23.88	3	/	/	/	/	23	34.19	Negative
P5	F	24	18.37	2	/	/	/	/	25	31.93	Negative
P6	M	51	22.49	3	/	Yes	/	/	28	31.77	Negative
P7	M	45	21.25	2	/	/	/	/	14	33.36	Negative
P8	F	48	23.14	2	/	/	/	/	24	33.65	Negative
P9	M	56	29.05	3	/	/	/	/	11	30.57	Negative
P10	F	25	16.00	3	/	Yes	/	Yes	9	30.12	Negative
P11	M	31	25.06	3	/	/	/	/	20	29.77	Negative
P12	F	32	22.43	3	/	Yes	/	/	10	29.45	Negative
P13	F	54	24.11	3	/	/	/	/	12	32.39	Negative
P14	M	67	24.21	/	Yes	/	/	/	7	30.27	Negative
P15	M	65	21.80	3	Yes	/	/	Yes	7	27.04	Negative
P16	F	28	22.77	2	/	Yes	/	/	7	33.61	Negative
P17	M	35	19.49	2	/	/	Yes	/	15	32.15	Negative
P18	F	23	19.31	3	/	/	/	/	17	33.04	Negative
P19	F	24	27.55	3	/	/	/	/	25	33.00	Negative
P20	M	66	18.42	/	Yes	Yes	/	/	7	30.30	Negative
P21	M	33	21.56	2	/	/	/	/	8	31.92	Negative
P22	M	43	26.95	3	Yes	/	/	/	35	26.53	Negative

## Discussion

As an imperative public health issue, the phenomenon of recurrently positive nucleic acid test in patients with SARS-CoV-2 has raised considerations of researchers, residents and policy makers worldwide (12, 15, 16). However, “recurrently positive” is a rather vague term, and its causes still remain to be elucidated. Beyond doubt, *de novo* reinfection is one of the plausible explanations, as evidenced by the detection of phylogenetically distinct genomic sequence in the first and second infection episodes in one symptomatic RT-PCR re-positive case (13). Debatably, Young et al. argued that the recurrently positive patients only contains non-infectious virus genomic fragments, which may intermittently or continuously secret low-level of viral RNA, leading to fluctuated RT-PCR results (30). Some others believe that the hospital discharge based on false negative readings may be another cause of seemingly re-positivity at later timepoints, including but not limited to the undetected virus in the lung by regular sampling means; insufficient amount of specimen; non-standard sample transportation and laboratory errors (31, 32). Therefore, this study was intended to address the unmet needs of this multifactorial phenomenon, providing evidence on whether patients re-experiencing positive nucleic acid test could have infectious potentials and need to be quarantined. We also strived to propose machine learning algorithms to screen out subjects who would be safe on self-monitoring.

The major characteristics of the recruited subjects were mostly young to middle-aged, mildly symptomatic and well vaccinated. We demonstrated that a multitude of recruited subjects promptly reached negative results on the first two nucleic acid tests after admission, suggesting quarantine may be redundant in these patients. However, we did aware that a small proportion demonstrated delayed and labile Ct value restoration, simultaneously with the more frequent report of close contacts infection during recurrence, indicating that these subjects may need quarantine.

In conformity with the grouping based on the first two Ct values after admission, the two trajectories identified by the GBTM analysis perfectly resembled the features of the rapid and delayed recovery groups, confirming that the latter has a significantly lower Ct value at recurrence, with more symptoms and comorbidities, and could pose a threat to infect the others according to our epidemiological survey. In order to pick out those with delayed recovery features for quarantine, we developed machine learning algorithms, using only five simple indices, to predict the Ct value recovery patterns after recurrence with high performance. With the proposed calculator, healthcare-professionals are able to efficiently and feasibly differentiate individuals who needs to quarantine and who can be put on self-monitoring.

Unfortunately, negative virus isolation was reported for all selected samples, even in those two reported close contacts infection. Exiting evidence suggests that a positive RT-PCR test result does not necessarily translate to infectivity, as it fails to distinguish viral replication from non-infectious nucleic acid residues. When the viral RNA concentration under 5.4 log_10_ copies/ml, there only less than 5% successful rate in viral isolation (33). Yang et al. revealed that 96% of recurrently positive patients had a maximum viral concentration of <5 log_10_ copies/ml (30). As a result, consistently, several studies have reported negative outcomes in virus isolation in recurrently positive patients, indicating that the rebound Ct values is likely to be the amplification of dead virus remaining during RT-PCT, rather than reinfection or reactivation of virus (30, 34, 35). Together, these evidence further support our hypothesis that most of the patients with re-positivity have a low transmission risk and quarantine can only be reserved for the suspicious cases as discriminated by the predictive model.

There are some limitations to consider when interpreting the results. To begin with, as a single-centered study, our analysis could be subjected to potential bias, even with a robust sample size. However, it should be noted that ours was the biggest shelter hospital in Shanghai during the studying period, and was the main designated hospital to treat recurrently positive patients from all areas of the city. Secondly, this study primarily centered on subjects aged between 16 and 80, without severe symptoms. Thus, the analysis and proposed predictive model may not be applied to children and those with recurrently critical SARS-CoV-2 infection. Furthermore, virus isolation was only done at a single timepoint in randomly selected subjects. Plus, the success rate could be compromised by several uncertainties during transportation, preservation and sample handling. Although with standard technique and seasoned experience in virologic studies, the negative outcomes in virus isolation should still be interpreted with caution, as sporadic recurrently cases with culturable virus have been reported (13, 14). Herewith, these ambiguities highlight the usefulness of applying our predictive model to discriminate the suspiciously contagious individuals to quarantine.

In conclusion, quarantine in shelter hospitals seems to be unnecessary for a substantial proportion of patients experiencing recurrently positive nucleic acid test after initial recovery from SARS-CoV-2 infection, as evidenced by their mild symptoms, rapid Ct value recovery and negative virus isolation results. However, attentions must be paid to those with delayed Ct value restoring trace, who tend to have comorbidities, persistent cough symptoms, and are likely to be infectious based on the epidemiological investigations, even though affirmative virologic evidence is lacking at the present stage. To assist the selection of the suspiciously infectious individuals for quarantine, we further proposed machine learning models using 5 simple indices, and achieved high predictive performance. The outcomes from this study may provide useful evidence for the enaction of SARS-CoV-2 pandemic prevention strategies regarding recurrently positive patients in the future.

## Data availability statement

The raw data supporting the conclusions of this article will be made available by the authors, without undue reservation.

## Ethics statement

The studies involving human participants were reviewed and approved by the Ethics Committee Boards of Ren Ji Hospital. The patients/participants provided their written informed consent to participate in this study.

## Author contributions

Q-XS and JZ designed the study, vouch for the integrity, and accuracy of the data. ZJ and WF assisted the revision of the study protocol. Q-XS, WF, CZ, CP, MCh, XZ, WZ, JW, MCa, SW, and LP participated in the assessment, sampling, and data collection. ZJ, CZ, and CP conducted the statistical analysis and developed the predictive algorithms. XC performed the virus isolation experiments. Q-XS and ZJ interpreted the results and drafted the manuscript. QX and JZ critically revised the manuscript. All authors have approved the final version of the manuscript.

## Conflict of interest

The authors declare that the research was conducted in the absence of any commercial or financial relationships that could be construed as a potential conflict of interest.

## Publisher's note

All claims expressed in this article are solely those of the authors and do not necessarily represent those of their affiliated organizations, or those of the publisher, the editors and the reviewers. Any product that may be evaluated in this article, or claim that may be made by its manufacturer, is not guaranteed or endorsed by the publisher.

## Supplementary material

Supplementary Figure 1 The percentage of overall and each detailed symptom during the initial infection and at recurrence.

Supplementary Table 1 Comparisons of abnormal rate of each laboratory test parameter in patients between the two trajectory groups. ^#^The comparisons between patients in group 1 and group 2. ^*^Indicates statistically significant difference.

Supplementary Table 2 Detailed parameters of the machine learning models. AUC, area under the curve; CI, confidence interval; LR, logistic regression; NNET, neural network; NPV, negative predictive value; PPV, positive predictive value; RAW, the predictive model using cycle threshold value at recurrence only; RF, random forest regression.
