# Supplementary figures and images for: The machine learning model based on trajectory analysis of ribonucleic acid test results predicts the necessity of quarantine in recurrently positive patients with SARS-CoV-2 infection
Source: Front Public Health. 2022 Nov 17;10:1011277. doi: 10.3389/fpubh.2022.1011277 (PMC9714505; doi:10.3389/fpubh.2022.1011277)

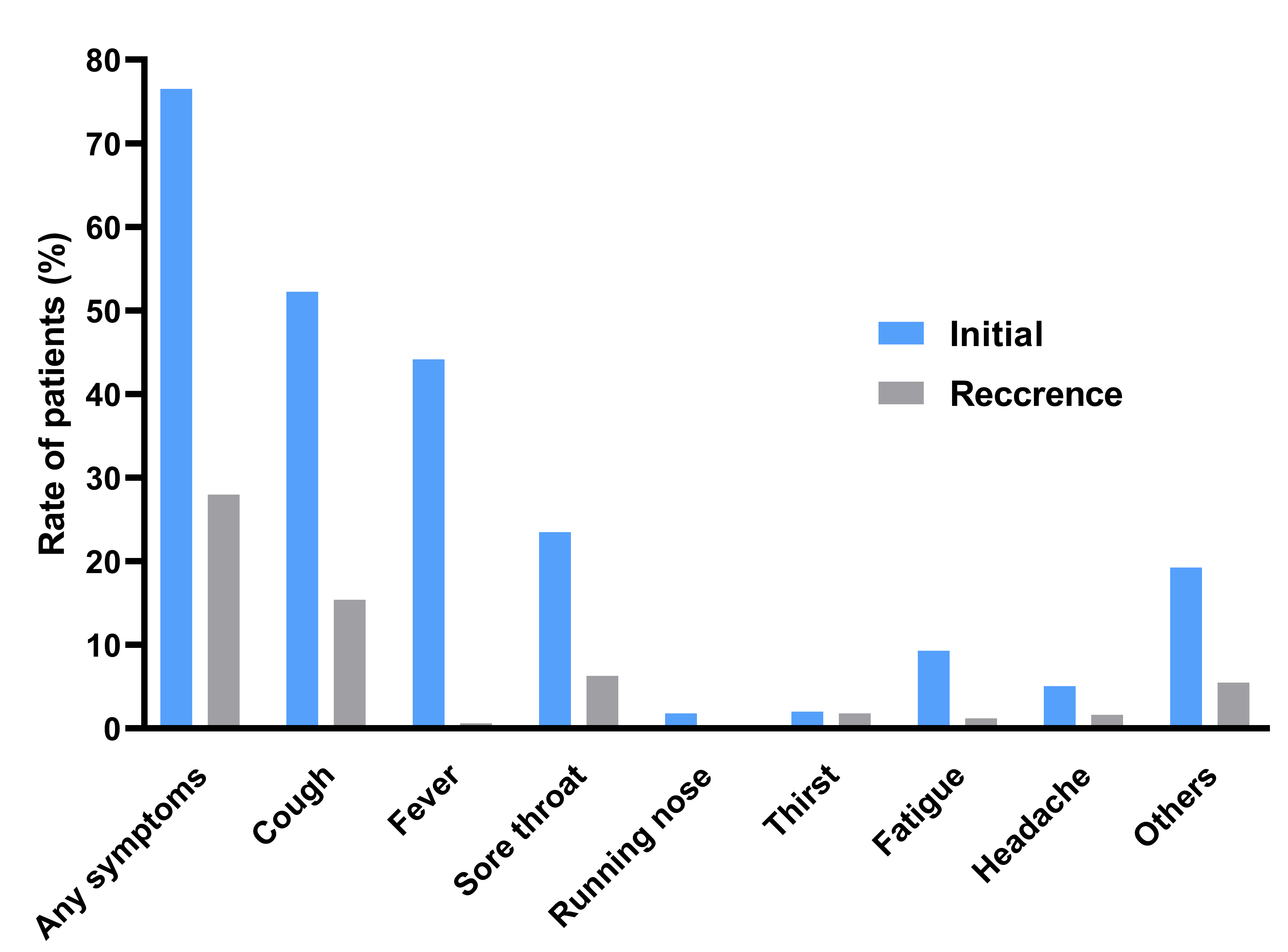

Supplement: Supplementary Figure 1 — The percentage of overall and each detailed symptom during the initial infection and at recurrence. [file Image_1.TIF]
