# Supplementary material for: The machine learning model based on trajectory analysis of ribonucleic acid test results predicts the necessity of quarantine in recurrently positive patients with SARS-CoV-2 infection
Source: Front Public Health. 2022 Nov 17;10:1011277. doi: 10.3389/fpubh.2022.1011277 (PMC9714505; doi:10.3389/fpubh.2022.1011277)
Supplement: Supplementary Table 1 — Comparisons of abnormal rate of each laboratory test parameter in patients between the two trajectory groups. #The comparisons between patients in group 1 and group 2. *Indicates statistically significant difference. [file Table_1.DOCX]

**Supplementary table 1. Comparisons of abnormal rate of each laboratory test parameter in patients between the two trajectory groups**

| **Variables** | **Reference value** | **Units** | **Total (n=288)** | **Group 1 (n=260)** | **Group 2 (n=28)** | **P value^#^** |
| --- | --- | --- | --- | --- | --- | --- |
| Platelet count | 125-350 | x10^9/L | 21(7.29%) | 18(6.92%) | 3(10.71%) | 0.4417 |
| Platelet ratio | 0.178-0.38 | % | 30(10.42%) | 28(10.77%) | 2(7.14%) | 0.7506 |
| Hemoglobin | 130-175 | g/L | 67(23.26%) | 61(23.46%) | 6(21.43%) | 0.8088 |
| Absolute value of neutrophil | 1.8-6.3 | x10^9/L | 21(7.29%) | 19(7.31%) | 2(7.14%) | 1.0000 |
| Percentage of neutrophil | 40-75 | % | 13(4.51%) | 11(4.23%) | 2(7.14%) | 0.3664 |
| Absolute value of eosinophil | 0.02-0.32 | x10^9/L | 27(9.38%) | 23(8.85%) | 4(14.29%) | 0.3143 |
| Percentage of eosinophil | 0-1 | % | 10(3.47%) | 7(2.69%) | 3(10.71%) | 0.0622 |
| Absolute value of basophil | 0-0.06 | x10^9/L | 9(3.13%) | 9(3.46%) | 0(0.00%) | 1.0000 |
| Percentage of basophil | 0.4-8 | % | 6(2.08%) | 5(1.92%) | 1(3.57%) | 0.4617 |
| Mean platelet volume | 8.0-12.5 | fl | 11(3.82%) | 10(3.85%) | 1(3.57%) | 1.0000 |
| Mean corpuscular hemoglobin concentration | 316-354 | g/L | 7(2.43%) | 7(2.69%) | 0(0.00%) | 1.0000 |
| Mean corpuscular hemoglobin | 27-34 | pg | 15(5.21%) | 15(5.77%) | 0(0.00%) | 0.3761 |
| Mean corpuscular volume | 82-100 | fl | 20(6.94%) | 19(7.31%) | 1(3.57%) | 0.7044 |
| Hematokrit | 40-50 | L/L | 111(38.54%) | 100(38.46%) | 11(39.29%) | 0.9322 |
| Erythrocyte count | 4.3-5.8 | x10^12/L | 68(23.61%) | 61(23.46%) | 7(25.00%) | 0.8555 |
| Red blood bell distribution width-SD | 37-54 | fl | 2(0.69%) | 2(0.77%) | 0(0.00%) | 1.0000 |
| Red blood cell distribution width-CV | 11.5-16.0 | % | 12(4.17%) | 12(4.62%) | 0(0.00%) | 0.6144 |
| Absolute value of monocyte | 0.1-0.6 | x10^9/L | 26(9.03%) | 22(8.46%) | 4(14.29%) | 0.2980 |
| Percentage of monocyte | 3-10 | % | 5(1.74%) | 1(0.38%) | 4(14.29%) | 0.0003^*^ |
| Platelet large cell ratio | 19.1-47.0 | % | 37(12.85%) | 33(12.69%) | 4(14.29%) | 0.7683 |
| White blood cell count | 3.5-9.5 | x10^9/L | 19(6.60%) | 17(6.54%) | 2(7.14%) | 1.0000 |
| Total bilirubin | 0-23 | umol/L | 17(5.90%) | 14(5.38%) | 3(10.71%) | 0.2215 |
| Aspartate aminotransferase | 15-40 | U/L | 38(13.19%) | 33(12.69%) | 5(17.86%) | 0.3908 |
| Alkaline phosphatase | 45-125 | U/L | 17(5.92%) | 13(5.02%) | 4(14.29%) | 0.0708 |
| Alanine aminotransferase | 9-50 | IU/L | 78(27.08%) | 67(25.77%) | 11(39.29%) | 0.1262 |
| Albumin | 40-55 | g/L | 16(5.57%) | 15(5.79%) | 1(3.57%) | 1.0000 |
| γ-Glutamyltransferase | 10-60 | U/L | 50(17.42%) | 45(17.37%) | 5(17.86%) | 1.0000 |
| Creatinine | 57-97 | umol/L | 76(26.39%) | 69(26.54%) | 7(25.00%) | 0.8607 |
| eGFR-EPI Cr | >=90 | ml/min/1.73m2 | 24(8.33%) | 23(8.85%) | 1(3.57%) | 0.4881 |
| Absolute natural killer cells (CD3- CD16+CD56+) | 220-735 | cells/uL | 74(25.78%) | 66(25.48%) | 8(28.57%) | 0.7226 |
| Natural killer cells (CD3-CD16+CD56+) | 8.7-38.3 | % | 22(7.67%) | 18(6.95%) | 4(14.29%) | 0.2491 |
| Absolute value of Lymphocytes | 0.8-4 | x10^9/L | 22(7.64%) | 20(7.69%) | 2(7.14%) | 1.0000 |
| Percentage of lymphocytes | 20-50 | % | 19(6.60%) | 17(6.54%) | 2(7.14%) | 1.0000 |
| Absolute value of T lymphocytes (CD3+ CD19-) | 948-1943 | cells/uL | 72(25.35%) | 62(24.12%) | 10(37.04%) | 0.1423 |
| Absolute value of T lymphocytes (CD3+ CD19-) | 52.4-81.4 | % | 34(11.97%) | 29(11.28%) | 5(18.52%) | 0.3434 |
| Ts lymphocyte absolute values (CD3+ CD8+) | 299-882 | cells/uL | 60(21.13%) | 53(20.62%) | 7(25.93%) | 0.5208 |
| Ts lymphocytes (CD3+ CD8+) | 11.7-40.3 | % | 17(5.99%) | 15(5.84%) | 2(7.41%) | 0.6693 |
| Absolute value of Th Lymphocytes (CD3+CD4+) | 447-1030 | cells/uL | 65(22.89%) | 59(22.96%) | 6(22.22%) | 0.9311 |
| Th Lymphocytes (CD3+CD4+) | 23.9-46.3 | % | 35(12.32%) | 29(11.28%) | 6(22.22%) | 0.1198 |
| Absolute value of B lymphocytes (CD3-CD19+) | 102-443 | cells/uL | 42(14.79%) | 39(15.18%) | 3(11.11%) | 0.7776 |
| Absolute value of B lymphocytes (CD3-CD19+) | 4.7-19.3 | % | 37(13.03%) | 32(12.45%) | 5(18.52%) | 0.3690 |
| CD4/CD8 ratio | 0.8-3.2 | - | 27(9.51%) | 26(10.12%) | 1(3.70%) | 0.4893 |
| Tumor necrosis factor-α | 0-4.6 | pg/ml | 9(3.14%) | 9(3.47%) | 0(0.00%) | 0.6069 |
| Interleukin-8 | 0-20.6 | pg/ml | 1(0.35%) | 1(0.39%) | 0(0.00%) | 1.0000 |
| Interleukin-6 | 0-5.30 | pg/ml | 16(5.63%) | 13(5.06%) | 3(11.11%) | 0.1856 |
| Interleukin-5 | 0-3.1 | pg/ml | 1(0.35%) | 1(0.39%) | 0(0.00%) | 1.0000 |
| Interleukin-4 | 0-3.0 | pg/ml | 19(6.69%) | 18(7.00%) | 1(3.70%) | 1.0000 |
| Interleukin-2 | 0-5.71 | pg/ml | 3(1.06%) | 3(1.17%) | 0(0.00%) | 1.0000 |
| Interleukin-1β | 0-12.4 | pg/ml | 2(0.70%) | 2(0.78%) | 0(0.00%) | 1.0000 |
| Interleukin-12p70 | 0-3.4 | pg/ml | 6(2.11%) | 6(2.33%) | 0(0.00%) | 1.0000 |
| Interleukin-10 | 0-4.91 | pg/ml | 8(2.82%) | 7(2.72%) | 1(3.70%) | 0.5550 |
| γ-Interferon | 0-7.42 | pg/ml | 2(0.70%) | 2(0.78%) | 0(0.00%) | 1.0000 |
| α-Interferon | 0-8.5 | pg/ml | 3(1.06%) | 2(0.78%) | 1(3.70%) | 0.2598 |
| Parathyroid hormone | 12-72 | pg/ml | 21(7.39%) | 18(7.00%) | 3(11.11%) | 0.4340 |
| 25 hydroxyvitamin D | 11.1-42.9 | ng/ml | 22(7.75%) | 20(7.78%) | 2(7.41%) | 1.0000 |
| Procalcitonin | >=0.1 | ng/ml | 1(0.35%) | 1(0.39%) | 0(0.00%) | 1.0000 |
| C-reaction protein | 0-8 | mg/L | 14(4.86%) | 10(3.85%) | 4(14.29%) | 0.0363^*^ |
| Serum amyloid A | 0-10.08 | mg/L | 16(5.57%) | 11(4.23%) | 5(18.52%) | 0.0107^*^ |
| Fibrin/fibrinogen degradation products | <5 | ug/ml | 4(1.40%) | 4(1.55%) | 0(0.00%) | 1.0000 |
| D-dimer | 0-0.5 | DDU ug/ml | 11(3.86%) | 9(3.50%) | 2(7.14%) | 0.2950 |
| Glucose | 3.9-6.1 | mmol/L | 126(44.06%) | 114(44.02%) | 12(44.44%) | 0.9659 |
