# Supplementary material for: The machine learning model based on trajectory analysis of ribonucleic acid test results predicts the necessity of quarantine in recurrently positive patients with SARS-CoV-2 infection
Source: Front Public Health. 2022 Nov 17;10:1011277. doi: 10.3389/fpubh.2022.1011277 (PMC9714505; doi:10.3389/fpubh.2022.1011277)
Supplement: Supplementary Table 2 — Detailed parameters of the machine learning models. AUC, area under the curve; CI, confidence interval; LR, logistic regression; NNET, neural network; NPV, negative predictive value; PPV, positive predictive value; RAW, the predictive model using cycle threshold value at recurrence only; RF, random forest regression. [file Table_2.DOC]

| **Supplementary table 2. Detailed parameters of the machine learning models** | | | | | | |  |
| --- | --- | --- | --- | --- | --- | --- | --- |
| Model | Sensitivity | Specificity | PPV | NPV | AUC | 95%CI |  |
| LR | 0.796 | 0.842 | 0.965 | 0.432 | 0.844 | (0.767 - 0.903) |  |
| NB | 0.942 | 0.737 | 0.951 | 0.700 | 0.876 | (0.805 - 0.929) |  |
| NNET | 0.932 | 0.632 | 0.932 | 0.632 | 0.815 | (0.734 - 0.879) |  |
| RAW | 0.952 | 0.632 | 0.933 | 0.632 | 0.829 | (0.751 - 0.891) |  |
|  | | | | | | |  |
